# 200 years of dinosaur discoveries –a visual portrayal of their geographic and historical origins

**DOI:** 10.7717/peerj.21351

**Published:** 2026-05-27

**Authors:** Donald M. Henderson

**Affiliations:** Royal Tyrrell Museum of Palaeontology, Drumheller, Alberta, Canada

**Keywords:** Dinosaurs, History, Geography, Diversity, Graphical display

## Abstract

**Background:**

The first dinosaur to be formally named was *Megalosaurus* in 1824. Over the subsequent 200 years more than 1,200 different species of dinosaur have been discovered and named. The discoveries come from every continent, but different regions are characterized with different species from different geological time periods. Given this multi-generational collection history, from a wide variety of geographic areas, and representing a huge diversity of animals, it was felt that a new type of visual summary of the current state of our knowledge of dinosaur diversity and discovery history would be much more informative and revealing than a simple list comprised of words and numbers.

**Methods:**

Using recently published, comprehensive surveys of all known Mesozoic, non-avian dinosaurs, a series of computer-generated, horizontal, stacked, bar-type graphs are used to graphically show how our knowledge of dinosaur diversity has developed over the past 200 years at the decade level. The graphs use clusters of coloured boxes, with individual boxes scaled to the numbers of dinosaurs from a region, to enable comparisons of counts of dinosaur discoveries from different regions within and between decades. The colours are used to identify the countries and regions on an accompanying global map. This analysis of the historical growth in our knowledge of dinosaur diversity is presented in two ways: at the high level of the clades Theropoda, Sauropodomorpha and Ornithischia; and at a lower one with many detailed monophyletic and paraphyletic sub-group levels.

**Results:**

As of end of 2025 this survey finds that there are 1,259 reliably known dinosaur species: 428 ornithischians, 366 sauropodomorphs and 465 theropods. From the entire 19th century only 58 dinosaur species can be viewed as having been discovered and still be considered valid today. These 19th century discoveries came from Europe and the United States of America with just three exceptions. Dinosaur discovery and naming rates were very low, typically no more than 10 per decade per major clade, until the 1970s when the decadal counts began to exceed the total 19th century counts for the first time. Discoveries from 1920s began to fully reveal to the true global extent of dinosaur occurrences. The early 2000s saw Argentina and China become the leading countries for new dinosaurs, but Europe and the United States continue to produce new discoveries up to the present.

## Introduction

Dinosaurs first came onto the public scene in the middle decades of the 19th century with a flurry of discoveries predominantly from England, France and Germany (Sues, 2012). Although the bones of large, prehistoric animals that would come to be known as dinosaurs were sporadically reported beginning in the 17th century (Delair & Sargeant, 2002), the first formally described and named dinosaur did not appear until 1824 with the carnivore *Megalosaurus* (Buckland, 1824). However, the recognition of dinosaurs as a distinct taxonomic group would have to wait a further two decades (Owen, 1842). Our knowledge of dinosaurs grew slowly over the remaining middle decades of the 19th century with modest additions principally from various western European countries and similar numbers from the United States. With the discovery of the rich fossil deposits in the American west beginning in the 1870s, our knowledge of the diversity of dinosaurs rapidly increased (Colbert, Gillette & Molnar, 2012). Furthermore, these new discoveries were of truly gigantic animals that dwarfed what had been known from Europe, and completely the captivated the public imagination (Cadbury, 2000).

Except for a brief pulse of ornithischian discoveries between 1910 and 1930 associated with the Canadian Dinosaur Rush (Spalding, 1999), there was a long quiet period spanning the last decade of the 19th century and the first six of the 20th with just a few dinosaur discoveries per year. However, the rate of dinosaur discoveries jumped dramatically beginning in the 1970s, reflecting a maturing of the sciences of biology, geology and palaeontology. Dinosaurs were becoming better understood as fossils from various places across the globe contributed to a substantial foundation of knowledge that could be used to motivate and better interpret new discoveries, thus accelerating the development of the dinosaur studies and its public visibility.

The present study was made possible for three reasons: (1) the publication of a new, data-rich and comprehensive compilation of recognized dinosaur species, (2) the inspiration from a novel graphical method of presenting combined geographical and historical data, and (3) the ability to write a computer program to automate the tabulation and graphical presentation of the temporal and geographic data for 1,259 dinosaur species. As well as providing a summary of the current state of known dinosaur diversity, the present work shows the historical development of the discovery, naming and classification of dinosaurs in a new and visual way.

## Materials and Methods

The publication of Jones (2026) is an up-to-date, encyclopedic and thorough tabulation of all currently accepted Mesozoic, non-avian dinosaurs as of early 2024, and was the principal data source for the present study. Additional new species for 2024 and 2025 were taken from Wikipedia: https://en.wikipedia.org/wiki/2024_in_archosaur_paleontology, https://en.wikipedia.org/wiki/2025_in_archosaur_paleontology.

For each taxon four types of data were taken from these sources: the full genus and species name, the year of the taxon’s description and naming, its systematic position, and its country of origin. The aim of the present study is to document the historical progression of our knowledge of the diversity of dinosaurs, so only the country or region where the species was *first* collected was tabulated.

For the purposes of the present study, a species with a revised identification is considered to be ‘known’ or ‘discovered’ at the latest date that the revised name was established—independent of whether it was a genus or species name change. Prior to the most recent, and presumably now correct naming, we can think of re-identified and re-named organisms as having been previously hidden and residing unknown inside an earlier name. As an example, Lydekker named ‘Titanosaurus australis’ in 1893 based on fossils found in Argentina. Powell (1992) renamed the specimen as *Neuquensaurus australis*. *Neuquensaurus* has been set at 1992 in the data set as it can be considered to have been hidden inside ‘*T. australis*’ until 1992. A similar situation exists for many dinosaur species and genera that were named in the early years of dinosaur studies.

Although Huxley (1870) and Marsh (1877) suggested some sort of common origin for birds and dinosaurs in the 19th century, the idea did not get a rigorous formal treatment until late in the 20th century. To facilitate comparisons between the ideas and tabulations of what constituted a dinosaur in the19th century and the first three-quarters of the 20th century, this study will exclude birds and other paravians crownward of *Archaeopteryx*. This is also consistent with the way most people think of dinosaurs. While technically correct, the idea that present-day, small, feathered, flying, beaked, often arboreal, chirping and singing archosaurs are related to *Apatosaurus* and *Triceratops* still takes some getting used to. Furthermore, and thinking ecologically, birds would seem to have little in common with the extinct, long-tailed, exclusively terrestrial (non-flying) animals where a large fraction of them weighed much more than the largest living bird—the ostrich.

The ‘Graphical Science’ section of the February 2025 edition of Scientific American presented a visual summary of all the people who have travelled into space along with when they travelled and their countries of origin (Moskowitz & Wolf, 2025). This summary was done with a horizontal form of bar graph with collections of boxes, grouped by decade, indicating counts of astronauts from different countries arranged on alternate sides of a zigzag line. The sequence of vertices of this horizontal, zigzag line were associated with a sequence of decades of space travel beginning with the first manned spaceflights in the 1960s. The collections of boxes documenting each decade’s space travellers were individually coloured to show either the traveller’s specific country of origin or a broader geographical region of origin. The same colours were used on a global map highlighting the geographic origins of the travellers. This compact and visually appealing method of using a horizontal, coloured bar graph, along with a similarly coloured global map, was the inspiration to document two hundred years of dinosaur discoveries in a visual way. Quoting Oscar Wilde, the present work can be viewed as an example of “Imitation is the sincerest form of flattery”.

Given that there are 1,259 species in the present sample of validly named dinosaurs, it would have been totally impractical to tabulate the country and decade data by hand and then individually draw the many, sorted and properly scaled boxes on many zigzag plots. Instead, with the dinosaur data in a standard format, it was possible to devise a computer program to trawl through the data with its various partionings and count how many dinosaur first-occurrences were associated with each country, combine the yearly data into decadal bins for each country, and then draw the decadal zigzag lines and associated boxes. The visual presentation of dinosaur data for a given decade was done by showing the number of dinosaurs associated with a country for the decade as a coloured box whose area is proportional to its count. The boxes were sorted into descending order by size with the largest box drawn first at the decadal vertex of the zigzag line and subsequent smaller boxes arranged as symmetrically as possible, but in descending order, about the main central box. In forming the zigzag lines, the lengths of the pairs of line segments bounding a collection of decade boxes were automatically scaled to the total number of species described within a particular decade.

The systematic positions of taxa are presented at two levels. The demarcations of these levels are based on generally accepted, current classifications of dinosaurs with the caveat that the systematics of dinosaurs is an ongoing process that will never be resolved to everyone’s satisfaction. The first presentation is a high level one consisting of a taxon’s broad classification as either Theropoda, Sauropodomorpha or Ornithischia. The second level is done as either monophyletic clades or paraphyletic subgroups within the major three clades with the aim of providing more detailed views of the history of discovery of smaller groups of closely related dinosaurs. Jones (2026) admits that his cladistic organization of the dinosaurian species at lower levels is a sometimes subjective compromise between the various competing phylogenetic hypotheses for various dinosaurian groups that have been developed over the past few decades. For the present study the decisions about sub-group boundaries and which animals they contain may also seem somewhat arbitrary, but it is NOT the purpose of the current project to provide a rigorous systematic analysis, and practical considerations were just as important for determining the partitioning of the taxa. The most important aspect of the present study is that it be as comprehensive as possible in terms of species documented. Given the nature of cladistic classification, it would not have been practical in all cases to use strictly monophyletic subgroups as some would contain excessively large numbers of species, so the use of paraphyletic groupings was frequently employed to partition taxa into manageable chunks. The delineation of a paraphyletic group often depended on how many species could be clearly displayed in sufficient detail on a relatively small zigzag plot on a printed page. As an example, the Ceratosauria was divided in two: a paraphyletic group that included Ceratosauridae, Noasaurinae and Elaphrosaurinae comprising 22 species and the monophyletic Abelisauridae comprised of 34. Full details of which animals comprise the various second level partionings can be found in the Supplementary Information.

**Table 1:** Countries, regions and continents where dinosaurs have been discovered. Indented names denote those countries that are subsumed under the header names of larger geographic regions to enable plotting of dinosaur-hosting regions on a small scale map.

Africa	Europe	South America
Algeria	Armenia	Brazil
Angola	Austria	Chile
Egypt	Belgium	Colombia
Lesotho	Croatia	Ecuador
Libya	Czechia	Uruguay
Madagascar	England	Venezuela
Malawi	France	
Morocco	Germany	United States
Niger	Hungary	
South Africa	Italy	West Asia
Tanzania	Netherlands	India
Tunisia	Norway	Pakistan
Zimbabwe	Poland	
	Portugal	Middle Asia
Antarctica	Romania	Kazakhstan
	Scotland	Kyrgyzstan
Argentina	Spain	Russia
	Sweden	Ukraine
Australia	Switzerland	Uzbekistan
	Wales	
Canada		East Asia
	Mexico	Japan
China		South Korea
	Mongolia	
Greenland		South Asia
		Laos
		Thailand

Ideally, every new species would have its country of origin presented on a zigzag plot and on the global map, but for practical reasons some of the countries were combined into broader geographical regions. Large countries, countries with exceptionally high dinosaur counts, or continents with just a handful of dinosaurs that would be easily visible on a small-scale, global map were kept separate *e.g.*, Argentina (large country, high count) and Greenland (continent, low count). Smaller countries, or those areas with very few dinosaur discoveries, were combined into geographic regions, *e.g.*, Thailand and Laos were lumped into the larger region defined here as “South Asia”. Table 1 shows the geographic breakdown that was used to both partition the discovery data and to define the colouring of the global distribution map. An exception to the combining of small or dinosaur-sparse countries into broad regions for display purposes was done with a separate display of all the 19th dinosaur discoveries in the top level of groups of Theropoda, Sauropodomorpha and Ornithischia. The modest numbers of dinosaurs named during this century, and their roughly equal split between European and United States origins, enabled the production of a full map of western Europe that could clearly show the individual countries and their dinosaur first-appearance discoveries in the 19th century.

## Results

**Figure 1:**
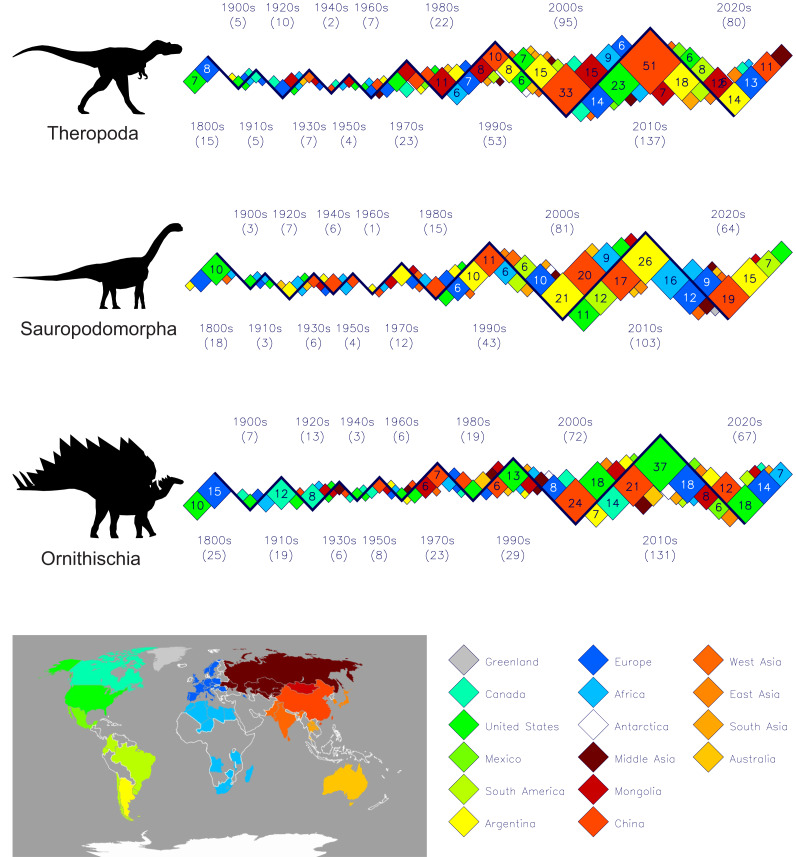
Plots of dinosaur discoveries by decade and by country or region for the past 200 years (up to 2025) partitioned across the three main dinosaur clades. Total numbers of taxa plotted per clade: Theropoda–465, Sauropodomorpha–366, Ornithischia–465. The coloured boxes represent the number of dinosaurs from a particular country or region (see map and associated legend) within a decade, and the area of each box is proportional to its dinosaur count. Numbers in parentheses adjacent to each decade name are the total counts for the decade. In the interests of legibility, only those boxes with counts of five or more are labelled. The smallest boxes on all three plots represent singletons. Four details stand out: (1) the long, quiet period from 1900 to 1970, (2) the abrupt doubling of new theropod and sauropod taxa in the 1990s, and new ornithischians in the 2000s, (3) the large numbers of saurischians from China and Argentina over the past 25 years, and (4) the large numbers of ornithischians from the United States over the same time period. See Supplementary Information for the data used to generate these plots (‘theropoda-all’, ‘sauropodomorpha-all’, and ‘ornithischia-all’) and Table 1 for details of the countries and regions named in the map legend. Silhouette identifications: Theropoda-*Albertosaurus libratus* based on illustration by M. Skrepnick in Currie (1997, Fig. 17.3), Sauropodomorpha–*Camarasaurus lentus* after Paul (1997), Ornithischia–*Stegosaurus stenops* after Paul (1987).

Figure 1 presents the decades and places of first-occurrence discoveries of all known dinosaurs for the past two hundred years partitioned across the three main clades of Theropoda, Sauropodomorpha and Ornithischia. See the Supplementary Information for the files ‘theropoda-all’, ‘sauropodomorpha-all’, and ‘ornithischia-all’ for the data used to generate these three zigzag plots. Some notable features revealed with these three plots are:

**Figure 2:**
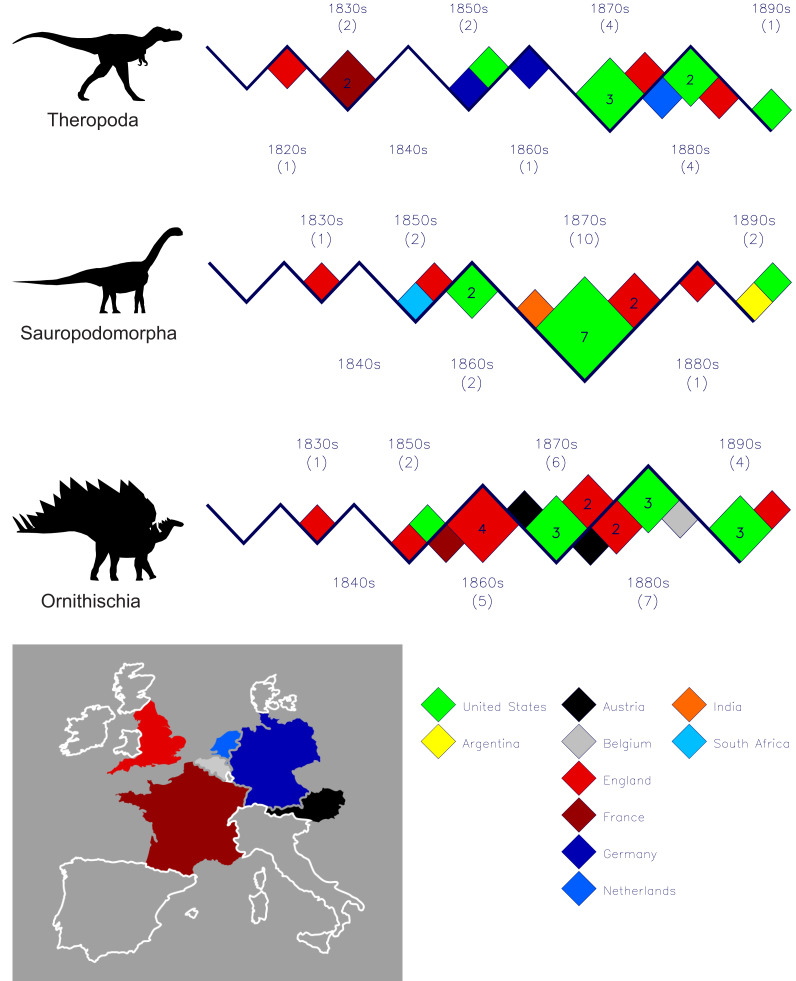
Plots of the dinosaur discoveries for the 19th century partitioned across the three main dinosaur clades. With the limited number of countries, it was possible to present a more detailed map of those European countries that had 19th century dinosaurian first occurrences. Except for the singleton sauropod discoveries from Argentina, India and South Africa, all 19th century discoveries were made in Europe and the United States (no map). Taxa counts per clade are: Theropoda–15, Sauropodomorpha–18, Ornithischia–25. The numbers in the boxes report the discovery counts for each country. Unnumbered boxes represent singletons. See Table 2 for full details of the data used to construct these plots. Silhouette identifications as per Fig. 1.

(1) The relatively tiny number of dinosaur species that are still considered to be valid that were discovered and named during the entire 19th century—Theropods 15, Sauropodomorphs 18, Ornithischians 25—when compared to the numbers from recent decades.
(2) The very low numbers of dinosaurs discovered during the years 1900 to 1970 for all three dinosaur groups. Especially depauperate are the sauropods with just three new species being reported in each of the first two decades of the 20th century, and just one(!) in the 1960s.
(3) The abrupt shift to the discovery of dinosaurs to a much wider geographic area than just the original 19th century European and United States localities. This begins in the 1910s with ornithischians from Canada and Africa, and in the 1920s with the discoveries of new theropods and sauropods from the southern and northern hemispheres (*e.g.*, Australia and Mongolia).
(4) The 1990s was the first decade when the numbers of new discoveries for all dinosaur groups exceeded those of the entire 19th century for the first time.
(5) The abrupt appearance of very large numbers of new dinosaurs from Argentina and China, beginning in the first decade of the 21st century. This was predicted 35 years ago by Dodson (1990).
(6) There has been a steady increase in numbers of discoveries per decade since the 1980s for all three clades. At the midpoint of the present decade, 2020–2029, it appears that this pattern will continue.

The modest number of 19th century dinosaur discoveries permits a complete and more detailed graphical depiction of their geographical origins, and this is done in Fig. 2. See Table 2 for a complete tabulation of 19th dinosaur discoveries and their countries. The most noticeable feature is the almost complete dominance by a few countries in Europe and the United States (see map on Fig. 2). Especially noticeable is relatively small England with its disproportionately high number of named dinosaurs when compared to neighbouring France. England has an area of approximately 130,000 km^2^, while France has an area of approximately 633,000 km^2^—roughly five times larger. However, 19th century England recorded three of the theropods (20%), six of the sauropodomorphs (28%), and 11 of the ornithischians (44%). During the same period, the much larger and immediately adjacent France recorded only two theropods (13%), none of the sauropods and only one of the ornithischians (5%).

**Table 2:** Nineteenth century dinosaur first occurrences organized by major clade and sorted by year of discovery.

**Theropoda**			**Ornithischia**		
*Megalosaurus bucklandii*	1824	England	*Hylaeosaurus armatus*	1833	England
*Streptospondylus altdorfensis*	1832	France	*Stenopelix valdensis*	1857	England
*Poekilopleuron bucklandii*	1836	France	*Hadrosaurus foulkii*	1858	United States
*Troodon formosus*	1856	United States	*Echinodon becklesii*	1861	England
*Compognathus longipes*	1859	Germany	*Scelidosaurus Harrisonii*	1861	England
*Archaeopteryx lithographica*	1861	Germany	*Acanthopholis horrida*	1867	England
*Aristosuchus pusillus*	1876	England	*Hypsilophodon foxii*	1869	England
*Dryptosaurus aquilunguis*	1877	United States	*Rhabdodon priscus*	1869	France
*Allosaurus fragilis*	1877	United States	*Struthiosaurus austriacus*	1871	Austria
*Coelurus fragilis*	1879	United States	*Dacentrurus armatus*	1875	England
*Betasuchus bredai*	1883	Netherlands	*Nanosaurus agilis*	1877	United States
*Ceratosaurus nasicornis*	1884	United States	*Stegosaurus stenops*	1877	United States
*Ornithodesmus cluniculus*	1887	England	*Anoplosaurus curtonotus*	1879	England
*Coelophysis bauri*	1889	United States	*Mochlodon suessi*	1881	Austria
*Ornithomimus velox*	1890	United States	*Iguanodon bernissartensis*	1881	Belgium
			*Polacanthus foxii*	1881	England
**Sauropodomorpha**			*Camptosaurus dispar*	1885	United States
			*Cumnoria prestwichii*	1888	England
*Thecodontosaurus antiquus*	1836	England	*Nodosaurus textilis*	1889	United States
*Pelorosaurus brevis*	1850	England	*Triceratops horridus*	1889	United States
*Massospondylus carinatus*	1854	South-Africa	*Claosaurus agilis*	1890	United States
*Anchisaurus polyzelus*	1865	United-States	*Torosaurus latus*	1891	United States
*Astrodon johnstoni*	1865	United-States	*Sarcolestes leedsi*	1893	England
*Ornithopsis hulkei*	1870	England	*Dryosaurus altus*	1894	United States
*Cetiosaurus oxoniensis*	1871	England			
*Titanosaurus indicus*	1877	India			
*Apatosaurus ajax*	1877	United States			
*Atlantosaurus montanus*	1877	United States			
*Camarasaurus supremus*	1877	United States			
*Dystophaeus viaemalae*	1877	United States			
*Amphicoelias altus*	1878	United States			
*Diplodocus longus*	1878	United States			
*Brontosaurus excelsus*	1879	United States			
*Dinodocus mackesoni*	1884	England			
*Barosaurus lentus*	1890	United States			
*Argyrosaurus superbus*	1893	Argentina			

Another noticeable feature from the 19th century is the abrupt appearances of significant numbers of dinosaurs in the 1870s and 1880s from the United States. This is in part a result of the opening up of the American west to settlers and explorations combined with the almost fanatical competition between the two main students of American dinosaurs at this time—O.C. Marsh and E. D. Cope (Colbert, 1968).

**Figure 3:**
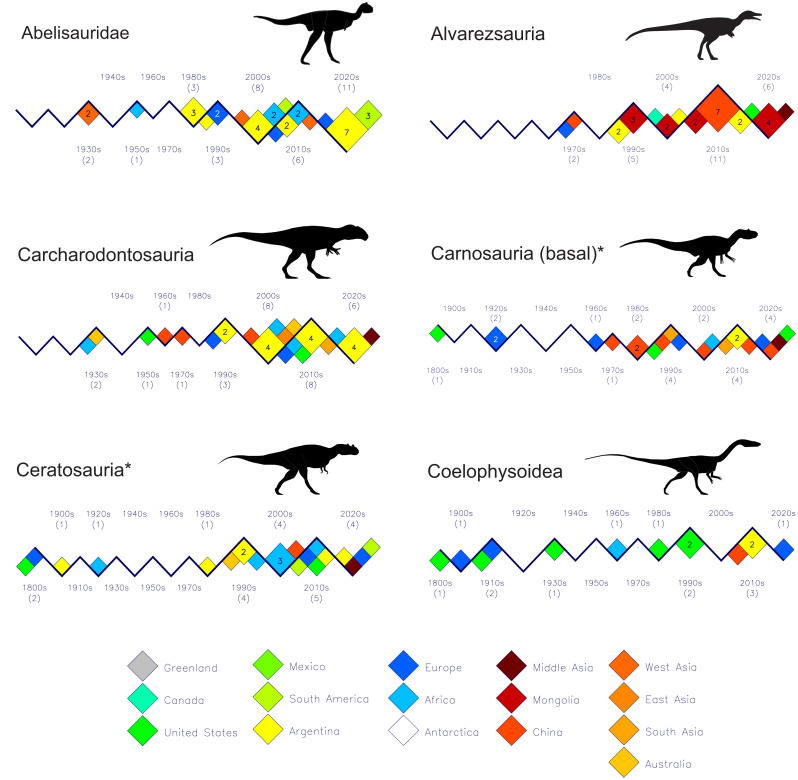
Plots of new theropod dinosaur discoveries by decade and by country or region for the past 200 years. Part I: Abelisaurids-Coelophysoids. Groups are organized alphabetically left-to-right and downwards. Group names marked with ‘*’ are paraphyletic. See Methods for the decisions and choices made to partition Theropoda into various clades and subgroups, and the file “theropoda-subgroups” in the Supplementary Information for the details of the taxa comprising the named groups. Two details stand out: (1) the abundance of abelisaurs and carcharodontosaurids from Argentina beginning in the first decade of the 21st century, and (2) the many new alvarezsaurids from Asia over the same time period. Silhouette identifications: Abelisauridae–*Carnotaurus* after Bonaparte, Novas & Coria (1990); Alvarezsauria–*Alvarezsaurus*- after https://fossil.fandom.com/wiki/Alvarezsaurus; Carcharodontosauria–*Carcharodontosaurus*–after Henderson & Nicholls (2015); Carnosauria (basal) *Allosaurus*–after Paul (1988); Ceratosauria–*Ceratosaurus*–after M. Skrepnick in Currie (1997, Fig. 17.3); Coelophysoidea–*Coelophysis*–after M. Skrepnick in Currie (1997, Fig. 17.3).

**Figure 4:**
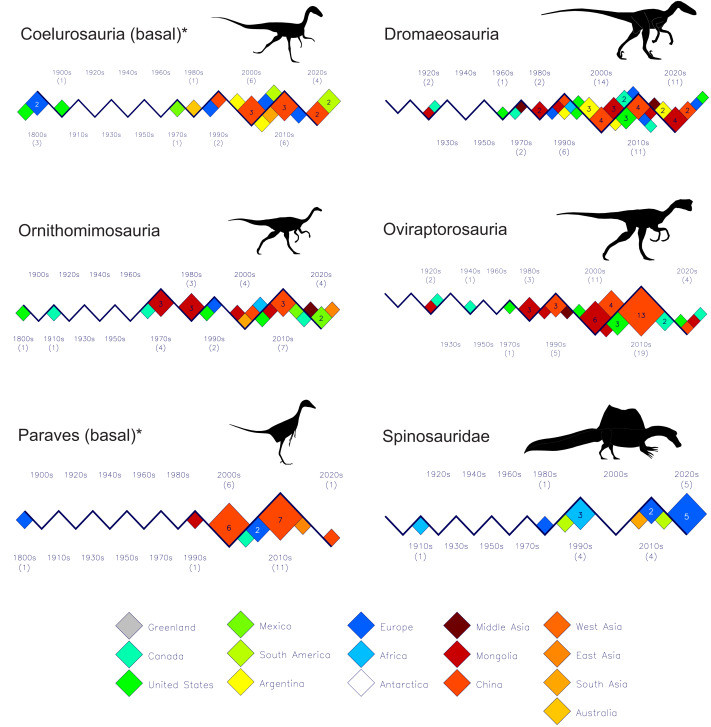
Plots of new theropod dinosaur discoveries by decade and by country or region for the past 200 years. Part II: Basal Coelurosauria–Spinosauridae. (1) The large numbers of small (<100 kg) theropods from Asia since the beginning of the 21st century, and (2) Europe as the dominant source of spinosaurids for the past two decades. Silhouette identifications: Coelurosauria (basal)–*Compsognathus* after Ostrom (1978); Dromaeosauria–*Deinonychus*, Ornithomimosauria–*Ornithomimus*, Oviraptorosauria–*Oviraptor* all based on illustrations by M. Skrepnick in Currie (1997, Fig. 17.3); Paraves (basal)–*Archaeopteryx* after Wellnhofer (2009); Spinosauridae–*Spinosaurus* after Ibrahim et al. (2020). See Fig. 3 caption for other details.

**Figure 5:**
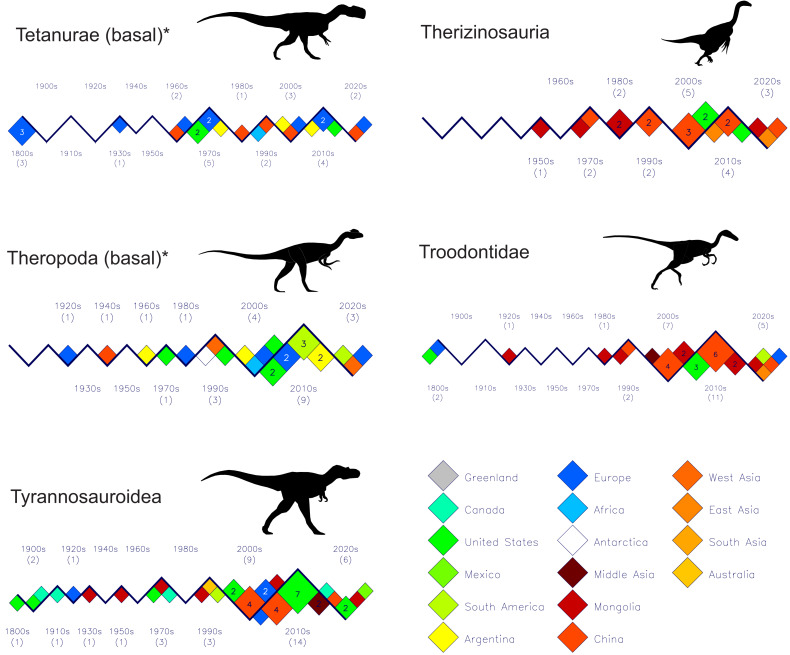
Plots of new theropod dinosaur discoveries by decade and by country or region for the past 200 years. Part III: Basal Tetanurae–Tyrannosauroidea. Note the large numbers of therizinosaurs and troodontids coming from Asia since the beginning of the 21st century and the substantial numbers of tyrannosaurs from the United States over the same time period. Silhouette identifications: Tetanurae (basal)–*Monolophosaurus* after Hartman (2003); Therizinosauria–*Therizinosaurus* after Paul (1997); Theropoda (basal)–*Dilophosaurus* after Welles (1984); Troodontidae–*Troodon* after Paul (1988); Tyrannosauroidea–*Albertosaurus* after illustration by M. Skrepnick in Currie (1997, Fig. 17.3). See Fig. 3 caption for other details.

Figures 3, 4 and 5 shows the discovery histories for the clades and subgroups that comprise the theropodan data set. See the file “theropoda-subgroups” in the Supplemental Information for the details of which species comprise each clade or subgroup. Perhaps the most notable feature is how China and Mongolia have been the principal sources of new coelurosaurs for the past 25 years. Also, although spinosaurids have generally been seen as a Gondwanan clade, the past 15 years shows seven of the nine new species coming from Europe (Fig. 4).

**Figure 6:**
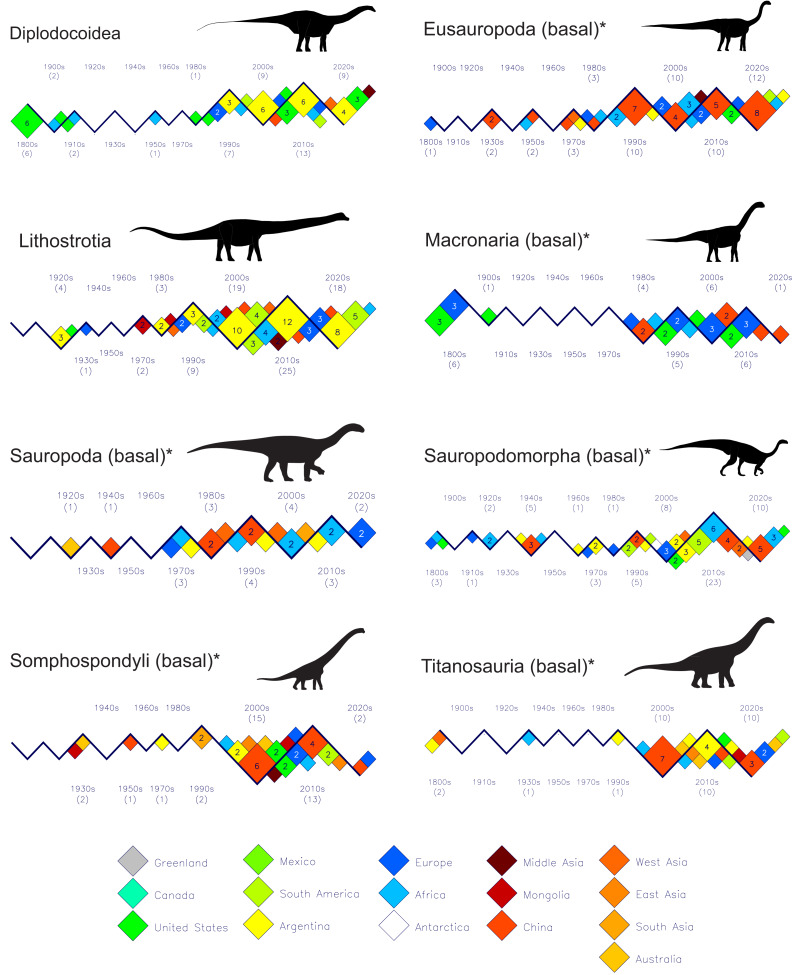
Plots of new sauropodomorph dinosaur discoveries by decade and by country or region for the past 200 years. (1) The meager sauropod record from 1800 to 1990 when compared to that of theropods and ornithischians for the same time period, (2) the large number of discoveries of diplodocoids and derived titanosaurs (Lithostrotia) from Argentina beginning in last decade of the 20th century, and (3) the minor contributions from the United States and Europe after their 19th century sauropod discoveries when compared to those of other regions. See the file “sauropodomorpha-subgroups” in the Supplemental Information for the details of the taxa comprising the named groups. Silhouette identifications: Diplodocoidea–*Apatosaurus* after Paul (1997); Eusauropoda (basal)–*Patagosaurus* after Paul (1997); Lithostrotia–*Patagotitan* after Carballido et al. (2017); Macronaria (basal)–*Camarasaurus* after Paul (1997); Sauropoda (basal)–*Gongxianosaurus* after https://www.deviantart.com/cisiopurple; Sauropodomorpha (basal)–*Plateosaurus* after Paul (1997); Somphospondyli (basal)–*Paluxysaurus* after Levi Bernardo, Creative Commons License; Titantosauria (basal)–*Dimantinasaurus* after https://www.deviantart.com/cisiopurple. See Fig. 3 caption for other details.

**Figure 7:**
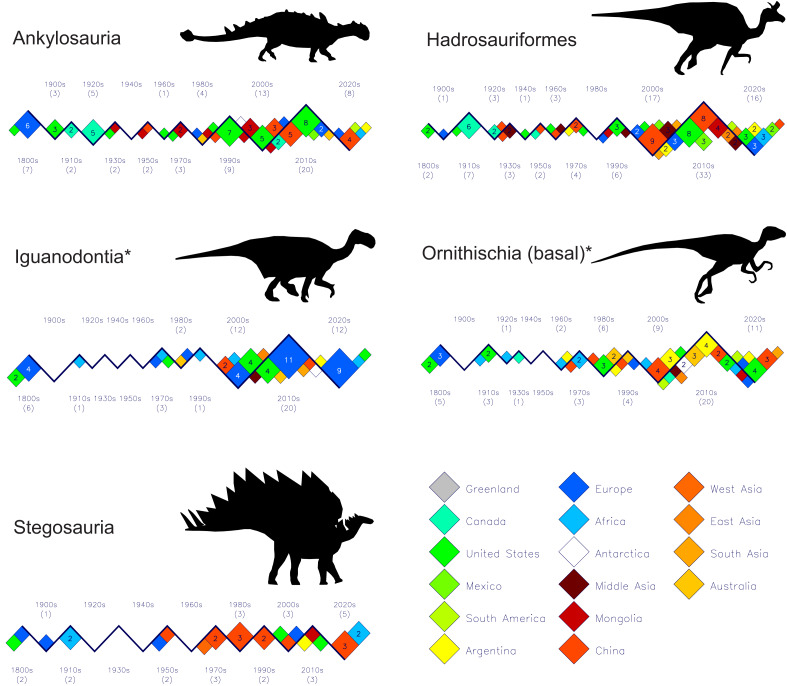
Plots of new ornithischian dinosaur discoveries, exclusive of Marginocephalia, by decade and by country or region for the past 200 years. (1) Hadrosauriformes come from a much wider geographic extent in the decades of the 21st century than they did in the previous centuries, and (2) after a long quiet period during the 20th century, Europe shows a resurgence with 24 new types of iguanodontians over the past 25 years. Silhouette identifications: Ankylosauria–*Euoplocephalus*; Hadrosauriformes–*Lambeosaurus*; Iguanodontia–*I. bernissartensis*; Ornithischia (basal)–*Heterodontosaurus*; Stegosauria–*S. stenops*. All figure silhouettes after Paul (1997).

Figure 6 presents the histories and geographies of discoveries of sauropodomorphan clades and subgroups. See the file “sauropodomorpha-subgroups” in the Supplemental Information for details of which species comprise each clade or subgroup. The first thing to note is that the sauropod fossil record was relatively meager up until the 1990s when compared with the records for the same time period for theropods (Figs. 3, 4 and 5) and ornithischians (Fig. 7). Secondly, the United States and Europe were the main sources of sauropod discoveries during the 19th century. However, over the past 125 years the few, recent contributions from these two regions have been completely overshadowed by contributions from elsewhere, with Argentina, China and African countries providing the vast majority of new discoveries. The only recent minor exceptions to this observation are the eight, new, basal macronarians from Europe from the past 30 years.

Figure 7 shows how ornithischians (exclusive of Marginocephalia) were principally known from European and North American localities with a scattering of African occurrences up until the 1930s. The known ranges of ornithischians began to expand rapidly beginning in the 1970s and 80s with China and Mongolia being important sources. One notable feature is the recent dramatic rise in the numbers of new iguanodontians from European countries with 24 being described in the past 25 years. See the file “ornithischia-subgroups” in the Supplemental Information for details of which species comprise each clade or subgroup.

**Figure 8:**
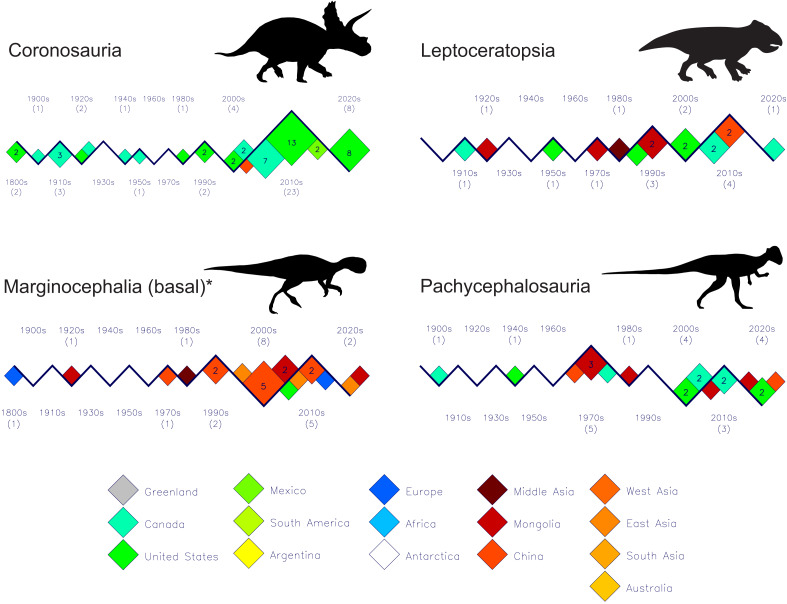
Decadal discovery plots for Marginocephalia. Except for the Pachycephalosauria, the new discovery numbers of the other three groups show reduced counts for the current decade and the decade-end tallies may not equal the discovery counts for the previous decade. Silhouette identifications: Coronosauria–*Triceratops*; Leptceratopsia–*Leptoceratops*; Marginocephalia (basal)–*Psittacosaurus*; Pachycephalosauria–*Stegoceras*. All figure silhouettes after Paul (1997).

The history of discoveries of the various marginocephalian subgroups are presented in Fig. 8. Their patterns of historical discovery rates and increasing geographic diversity mirror those of the other ornithischians presented in Fig. 7. However, they are from strictly northern hemisphere regions. Two notable features are (1) that only two basal forms are currently known from Europe, and (2) the almost exclusive limitation of coronosaurs to North America. The one exception to the latter observation is the Chinese *Sinoceratops zhuchengensis* (Xu et al., 2010). See the file “marginocephalia-subgroups” in the Supplemental Information for details of which species comprise each clade or subgroup.

**Figure 9:**
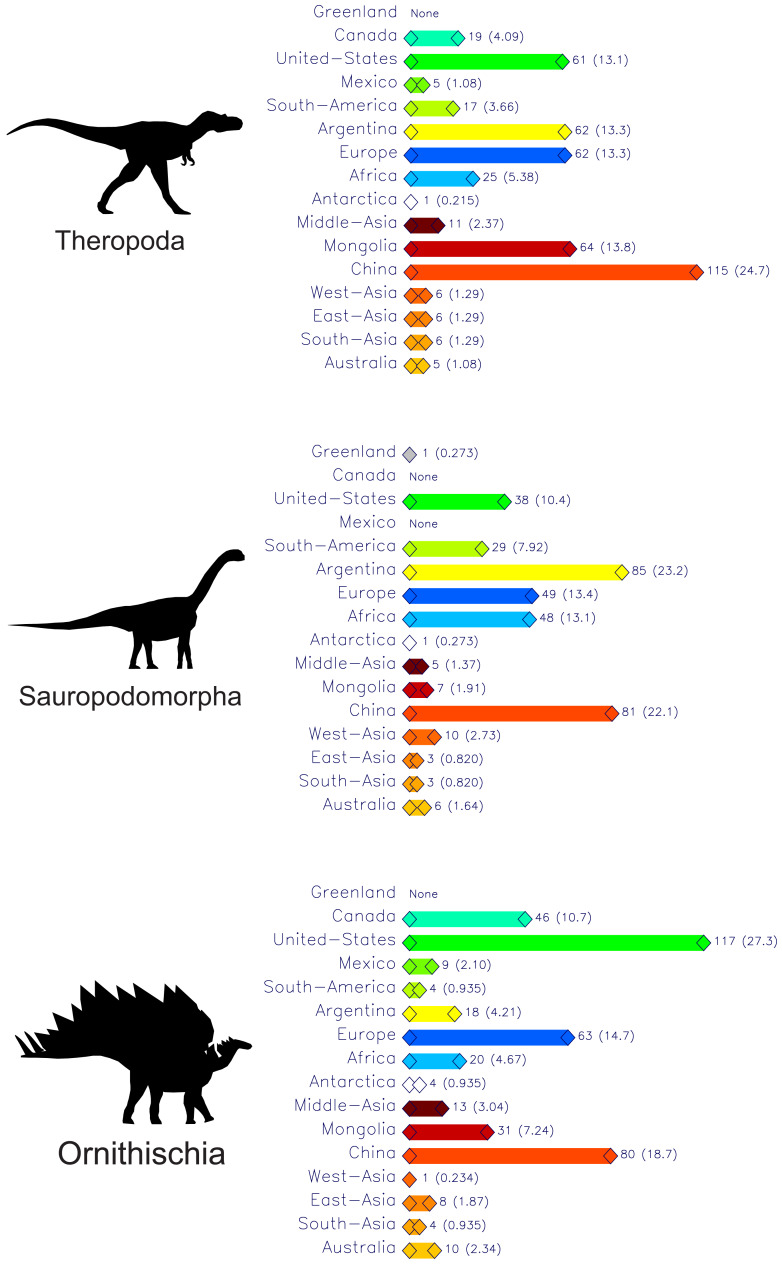
Summary plot showing the total numbers of dinosaur first-occurrences for each of the countries, regions or continents for the past 200 years for each of three main dinosaur clades. The pairs of numbers at the righthand ends of the coloured bars are the absolute counts for a region and in parentheses the percentage of the total for the given clade. Silhouette identifications as per Fig. 1.

Figure 9 is a summary of the total number of dinosaur discoveries for each of the origin regions defined for the present study. China is the leader in total with 276, followed by the United States with 216, and Europe with 174. Europe has the most balanced contribution with roughly equal contributions to the three main clades. China and the United States are biased towards theropods and ornithischians, respectively, while Argentina is the leader in sauropod discoveries, but with China close behind.

## Discussion

There is always a delay between when a fossil is discovered in the field (or possibly in a museum cabinet) and when its formal description and naming is finally published. As an example, the exceptionally well-preserved nodosaur *Borealopelta markmitchelli* was found in 2011 but required five years to prepare before being made public (Brown et al., 2017). Fortuitously, the year of actual discovery and the year of the publication of the description and naming of *Borealopelta* occurred in the same decade so no inaccuracy in the historical sequence at the decadal scale was introduced. Unfortunately, there will be instances of animals that are found in one decade, but not published on until many years, possibly decades, later, thus introducing inaccuracy in the historical sequence of discovery as reported here. One extreme example is that of the sauropod ‘*Gigantosaurus’ robusta* that was found in German East Africa in 1907 and named by Fraas (1908). However, improvements in our understanding of sauropods over the subsequent decades resulted in the specimen finally being assigned to a new genus and renamed as *Janenschia robusta* eighty three years later (Wild, 1991). One positive aspect of this delay is that after many decades of uncertainty, the later 1991 date for the identification of *Janenschia robusta* marks when we finally gained a proper understanding of the animal and its position within Sauropoda. Despite these dating problems, using the date of publication as the chronological marker for the historical first appearance of a new or revised taxon would appear to be the most parsimonious and objective strategy and would also be in keeping with the practices outlined in the ICZN (1999).

It is important to be aware that the use of cladistically defined names and relationships of dinosaurs in a historical study spanning 200 years is highly anachronistic when applied to discoveries from the decades preceding the 1980s. Modern thinking sees dinosaurs belonging to a larger group known as Archosauria (Benton, 2004). A version of the concept of Archosauria was first introduced by Cope (1869). Then a modified version, closer to the modern definition, that included dinosaurs, was used up until the 1970s (Benton, 2004). However, none of the authors of the older studies had the full modern view of the nature of the relationships between various dinosaur groups, nor a clear picture of the relationship of these animals to other reptiles, *e.g.* the position of dinosaurs within a larger group of reptiles now known as Diapsida. The first level of dinosaurian classification used in the present study—Theropoda, Sauropodomorpha and Ornithischia—was known, albeit in a simpler form, by the late 19th century. O.C. Marsh had recognized Theropoda and Sauropoda as distinct groups in Marsh (1881) and Marsh (1878), respectively, and a few years later the division between Saurischia (Theopoda+Sauropoda) and Ornithischia was recognized by Seeley (1887). For the next 100 hundred years various Linnean classification schemes were proposed, with varying degrees of success, to refine the memberships and interrelationships between the taxa of these three main divisions, and the position of dinosaurs within a wider evolutionary context. The application of cladistic methods to the systematics of dinosaurs began in the 1980s with the ornithischian study of Norman (1984) and the saurischian study of Gauthier (1986), and this soon led to a fundamental improvement in our understanding of the systematics of dinosaurs. See Benton (2004) for a comprehensive review.

Based on just the modest numbers of dinosaur discoveries of the 19th and the earliest 20th centuries, it appeared that certain dinosaur groups were limited to small geographic regions, eg. tyrannosauroids (Fig. 5) and coronosaurid marginocephalians (Fig. 8) were only known from the United States and Canada, while diplodocoid sauropods (Fig. 6) were only known from the United States during the 19th century. However, with the subsequent improvements to the dinosaurian fossil record it is clear that almost every dinosaur group had a global distribution. A notable exception is the Marginocephalia as they appear to have a strictly northern hemisphere range (Fig. 8).

19th century dinosaur discoveries came almost exclusively from Europe and the United States and this is not surprising as this is where modern science developed in concert with a need to explore and exploit the natural world to support the newly emerging industrial economies. There are just three exceptions to the above observation and all concern sauropodomorphs: *Argyrosaurus superbus* from Argentina, *Titanosaurus indicus* from India and *Massospondylus carinatus* from South Africa. However, these latter two countries were part of the British empire at the time and the naming and descriptions of their dinosaurs were first carried out by British researchers. *T. indicus* was named by Richard Lydekker in 1877 who was a Cambridge University graduate working in India (Chisholm, 1922) while *M. carinatus* was named by the famous Richard Owen in 1854 who was based at the Natural History Museum in London. It was also Lydekker who descirbed the Argentinian *Argyrosaurus* in 1893. It could be argued that these three seemingly “outside of Europe” 19th century discoveries were really just extensions of the prominence of dinosaur researchers in England at the time.

The high numbers of dinosaurs from 19th century England when compared to neighbouring and much larger France requires an explanation. This discrepancy could be the result of a difference in the availability of suitable outcrop for the discovery of terrestrial dinosaurs (Mannion et al., 2011). The Paris Basin is the major Mesozoic basin in France and accumulated terrestrial sediments starting in the Early Triassic. However, beginning in the Late Triassic and continuing for the remainder of the Mesozoic, the region was subject to marine conditions (Gély et al., 2014). This change in geography would have excluded terrestrial dinosaurs and reduced their likelihood of entering the fossil record in the region, thus lowering the numbers of dinosaurs reported from France.

Several studies suggest that we should not expect to find more than a few thousand dinosaur genera, with estimates of the total discoverable diversity being 1,200 (Dodson, 1990), 3,400 (Russell, 1995), 1,850 (Wang & Dodson, 2006) and 1,936 (Starrfelt & Liow, 2016). The number of genera and species documented in the present study—1,259—suggests that we are well past the halfway mark of total dinosaur genera to be discovered when the two most recent estimates are considered. A quantitative analysis of Wang & Dodson’s (2006) logistic function predicting cumulative future numbers of described dinosaur species reveals that we can expect the rate of dinosaur discoveries to start to decrease in 2037 (taking this to a ridiculously degree of over-precision, we can say the evening of the 17th of November, 2037. See Appendix for the derivation of this date and time). However, Wang & Dodson’s (2006) equation was based on discovery data from no younger than 2006, and before the abrupt increase in discoveries that came in the immediately following decade and half for all dinosaur groups (see Fig. 1). Another 15 years of dinosaur discoveries will be needed to see if the 2037 tipping point will occur. A repeat of the Wang & Dodson (2006) analysis using more recent data would be most interesting, but is beyond the scope of the present study.

## Conclusions

The third decade of 19th century saw the start of the formal scientific study of dinosaurs. Initially, rates of discovery were very low and geographically constrained with 55 of the 58 19th century discoveries coming from European countries or the United States of America. Dinosaur discovery rates continued to be low until the 1970s, with no more than 10 new species per decade per major clade (Theropoda, Sauropodomorpha, Ornithischia). With the cumulative improvements in our understandings of biology, geology and palaeontology during the 20th century our knowledge of dinosaur diversity expanded exponentially in the last quarter of that century. This exponential growth in knowledge is characterized by the recovery of more than 1,200 new dinosaurs from all continents and of new groups of dinosaurs that are unknown from the European or North American localities. Numbers of dinosaurs discovered per decade show increasing counts beginning in the 1980s and continuing up to the present.

## Supplemental Information

10.7717/peerj.21351/supp-1 Supplemental Information 1 List of all ornithischian species used; sorted by year

10.7717/peerj.21351/supp-2 Supplemental Information 2 Sauropodomorpha subgroup definitions

10.7717/peerj.21351/supp-3 Supplemental Information 3 List of all theropod species used; sorted by year

10.7717/peerj.21351/supp-4 Supplemental Information 4 Ornithischia subgroup definitions

10.7717/peerj.21351/supp-5 Supplemental Information 5 List of all sauropodomorphs used; sorted by year

10.7717/peerj.21351/supp-6 Supplemental Information 6 Theropod subgroup definitions

10.7717/peerj.21351/supp-7 Supplemental Information 7 Program used to generate zigzag plots for a specified group of dinosaurs species

10.7717/peerj.21351/supp-8 Supplemental Information 8 Collection of program subroutines to locate, draw and label boxes on a zigzag plot

## Appendix

Determining the rate of accumulation of new dinosaur genera.

Wang & Dodson (2006) presented the following logistic equation predicting the cumulative number of dinosaur discoveries, termed here as D, as a function of calendar years:

(1) \begin{eqnarray*}D \left( y \right) = \frac{1844}{1+\exp \nolimits (- \left( {\beta }_{0}-{\beta }_{1} \left( y-{\beta }_{2} \right) \right) )} \end{eqnarray*}

where ‘y’ is the year; *β*_0_, *β*_1_ and *β*_2_ are their preferred fitted parameters with values −1.05, 0.034 and 2006, respectively; and 1844 is their estimate of the total number of potentially discoverable genera. Equation (1) can be written in a more compact form if the exponential function in the denominator is expressed a separate function of ‘y’:

(2) \begin{eqnarray*}\beta \left( y \right) =\exp \nolimits (- \left( {\beta }_{0}-{\beta }_{1} \left( y-{\beta }_{2} \right) \right) )\end{eqnarray*}

Substituting $\beta \left( y \right) $ for the exponential function in the denominator of Eq. (1) gives the following:

(3) \begin{eqnarray*}D \left( y \right) = \frac{1844}{1+\beta \left( y \right) } \end{eqnarray*}

Taking the first derivative of Eq. (3) gives the following expression for the rate of change in dinosaur discoveries per year:

(4) \begin{eqnarray*} \frac{dD}{dy} = \frac{1844{\beta }_{1}^{2}(\beta { \left( y \right) }^{2}+\beta \left( y \right) )}{(1+\beta \left( y \right) )^{3}} \end{eqnarray*}

The year in which the rate of dinosaur discovery starts to decline can be found by equating Eq. (4) to 0 and solving for ‘y’. This gives a value of 2,036.882 for the year, and this decimal value can be expressed in the more familiar form of a calendar date as follows: 0.882 years equals 321.93 days where 321 days marks a date of November 17th. The remaining fraction of 0.93 days is the equivalent of 22.32 h, and 0.32 h is 19.2 min. The Wang and Dodson equation, Eq. (1) above, predicts that dinosaur discovery rates will begin their slow decline starting in the evening of November 17th, 2037 at approximately twenty past ten.

## Additional Information and Declarations

### Data Availability

The following information was supplied regarding data availability: The raw data is available in the Supplemental Files.
